# Development of a core outcome set (COS) and selecting outcome measurement instruments (OMIs) for non-valvular atrial fibrillation in traditional Chinese medicine clinical trials: study protocol

**DOI:** 10.1186/s13063-018-2904-0

**Published:** 2018-10-05

**Authors:** Ruijin Qiu, Min Li, Xiaoyu Zhang, Shiqi Chen, Chengyu Li, Hongcai Shang

**Affiliations:** Key Laboratory of Chinese Internal Medicine of Ministry of Education and Beijing, Dongzhimen Hospital, Beijing University of Chinese Medicine, Beijing, 100700 China

**Keywords:** Non-valvular atrial fibrillation, Traditional Chinese medicine, Core outcome set, Outcome measurement instrument

## Abstract

**Background:**

An increasing number of clinical trials of traditional Chinese medicine are being conducted in the treatment of non-valvular atrial fibrillation (NVAF) in China. However, the heterogeneity of outcomes and outcome measurement instruments has produced little evidence for traditional Chinese medicine in treating NVAF because many trials cannot be included in a meta-analysis. The majority of the trials did not report endpoint outcomes, side effects or other important outcomes for patients, which makes it difficult to evaluate the efficacy and safety of traditional Chinese medicine. Therefore, it is important to develop a core outcome set (COS). Although there are two related COSs for clinical trials of atrial fibrillation, the methodology is limited, and the perspectives of Chinese experts and patients are unclear. Therefore, we will develop a COS and recommend outcome measurement instruments after finishing the COS, which can be used for clinical trials of traditional Chinese medicine in NVAF.

**Methods/design:**

The method of the study will include eight stages led by a national multidisciplinary Steering Committee: (1) A systematic review will be developed to identify currently reported outcomes and traditional Chinese medicine syndromes in clinical trials of NVAF, (2) Semi-structured interviews of patients will be conducted to fill gaps in potential outcomes, (3) Traditional Chinese medicine syndrome names will be identified from medical records, (4) A dataset of traditional Chinese medicine syndrome names will be developed, (5) The investigation of traditional Chinese medicine syndromes will be conducted from cross-sectional study, (6) Two rounds of Delphi surveys will be carried out, (7) A consensus meeting will be conducted to develop a COS, and (8) Recommendations of outcome measurement instruments (OMIs), which should be used in the COS, will be developed.

**Discussion:**

The COS will improve the consistency of outcome reporting and reduce the reporting bias in NVAF clinical trials of traditional Chinese medicine to improve the value of traditional Chinese medicine clinical trials.

**Trial registration:**

This study is not a clinical trial, so it is registered in Core Outcome Measures in Effectiveness Trials Initiative (COMET). Registration number: 941. Registered on 22 December 2016.

**Electronic supplementary material:**

The online version of this article (10.1186/s13063-018-2904-0) contains supplementary material, which is available to authorized users.

## Background

Atrial fibrillation (AF) is one of the most common cardiac arrhythmias in China. The morbidity of AF is approximately 0.65%, of which non-valvular atrial fibrillation (NVAF) accounts for 65.2% [1]. AF is an age-related disease, and the morbidity is 0.1% per year in patients aged 40 years and older.

AF is related to the risk of all-cause mortality, cardiac mortality, sudden cardiac death, ischemic heart disease, stroke, heart failure, chronic kidney diseases, and peripheral arterial disease [2]. The therapeutic methods for treating AF include antiarrhythmic drugs, surgery, and catheter ablation, which have the adverse effects of proarrhythmia or are contraindicated [3].

Traditional Chinese medicine has developed its own unique principles and comprehensive theory and has played an indispensable role in health care for more than 2000 years in China [4, 5], and its practice is now popular worldwide [6]. Traditional Chinese medicine includes Chinese herbal medicine, dietary supplements, acupuncture, massage, moxibustion, cupping therapy, Tai Chi, *qigong*, and other exercise therapies [7]. Among them, Chinese herbal medicine formulas are the most common interventions in traditional Chinese medicine practice [4].

Syndrome differentiation is the basis of developing therapeutic principles in traditional Chinese medicine and includes the cause, nature, and location of pathologic changes at a certain stage of the disease [6], which can be comprehensively analyzed by clinical symptoms and signs through four methods of diagnosis: inspection, auscultation and olfaction, interrogation, and palpation.

Furthermore, the effectiveness of traditional Chinese medicine syndromes is one of the important outcomes in traditional Chinese medicine. Traditional Chinese medicine syndromes are formed by two elements: “location of the disease” and “features of the disease” [8]. Based on syndrome differentiation, Chinese herbal medicine treats specific syndromes of specific diseases and has the effect of holistic approaches and multitarget therapeutics, which is different from the single-target therapy of western medicine [7].

In China, a large number of patients have received traditional Chinese medicine. A national survey showed that 71.2% of patients preferred integrative medicine (integrated traditional Chinese medicine and Western medicine), and 18.7% of patients preferred traditional Chinese medicine in 2004 [9]. In addition, there has been an increasing number of clinical trials of traditional Chinese medicine in treating AF in recent years. However, there are some problems in these clinical trials, such as heterogeneous outcomes, surrogate outcomes, subjective outcomes, and composite outcomes, as well as the lack of endpoints or patient perspectives [10–12]. Meanwhile, few trials reported adverse effects or adverse events for traditional Chinese medicine treatment and the safety was unclear. In some cases, there is a lack of reports of outcome measurement instruments (OMIs), or the same outcomes were measured by different OMIs in different trials.

These issues may result in the inability of some clinical trials to conduct a meta-analysis in systematic reviews, the results of systematic reviews cannot translate into benefits for patients, or the effects of traditional Chinese medicine are exaggerated; consequently, the value of the clinical trials is reduced, and research investments are wasted to some extent.

To address these problems, developing a core outcome set (COS) and selecting OMIs for traditional Chinese medicine in clinical trials deserve consideration. A COS is an established set of outcomes that should be measured and reported, as a minimum set in all clinical trials in specific areas of health or health care [13]. The outcomes should be useful to different stakeholders, such as patients, clinicians, caregivers, and policymakers [14].

At present, there are two related COSs of AF included in the COMET (Core Outcome Measures in Effectiveness Trials) database. One was developed by the German Atrial Fibrillation Competence Network (AFNET) and the European Heart Rhythm Association (EHRA), which published in 2007 and can be used in clinical trials. The experts reached a consensus for required outcomes in seven relevant domains, including death, stroke, symptoms, quality of life, rhythm, left ventricular function, cost, and emerging outcome parameters [15]. The other was developed by the Heart Rhythm Society (HRS)/EHRA/the European Cardiac Arrhythmia Society (ECAS), which published in 2012. The experts recommended endpoints, such as time to recurrence of AF/flutter/tachycardia following ablation, or freedom from AF/flutter/tachycardia, for catheter and surgical ablation of AF in clinical trials [16].

However, the COS developed by EHRA in 2007 exhibited methodological flaws. First, the stakeholders included clinical experts, pharmaceutical industry representatives and researchers as stakeholders, but no patients were included. Second, while the COS achieved consensus in Europe, the perspectives from low- and middle-income countries were absent. Third, the COS was developed through consensus meetings without a systematic review to acquire an outcome list. Fourth, there have been no updated versions since 2007. Therefore, it is unclear if the COS is suitable for use in China. The COS developed by HRS/EHRA/ECAS is for catheter and surgical ablation; however, whether it can be used in clinical trials for drugs remains unclear. The most important flaw is that both COSs do not meet the needs of traditional Chinese medicine because there are no outcomes regarding traditional Chinese medicine syndromes. To summarize, it is necessary to development a COS that includes traditional Chinese medicine syndromes and achieves consensus between Chinese experts and patients in clinical trials of traditional Chinese medicine [17]. However, the morbidity of AF is low in patients aged under 50 years, and the traditional Chinese medicine syndromes are different between young and older individuals. In addition, in traditional Chinese medicine clinical trials of AF, Chinese herbal medicine is the most common intervention, which is effective in treating NVAF but not valvular atrial fibrillation. Meanwhile, there are few clinical trials for non-herbal therapy in treating AF (Additional file 1), and the results have little contribution to COSs, so the scope of the COS is developed for patients with NVAF who are aged 50–75 years old and accept Chinese herbal medicine treatment.

Selecting suitable and high-quality OMIs may reduce bias in drawing conclusions from trials, which cannot be ignored after developing a COS [18]. Diverse outcome reporting or a low quality of OMIs may result in a worthless investment and may be unethical for patients because patients undergo the risks of clinical trials but contribute little or nothing to the knowledge system [19]. Therefore, it is necessary to select a measurement instrument for each outcome included in a COS [20].

This study has two goals. The first goal is to provide a minimum outcome set that should be reported in NVAF patients who are aged 50–75 years old and receive Chinese herbal medicine in all types of clinical trials. The second goal is to select a measurement instrument for each outcome included in the COS with the method recommended by the COMET initiative after completing the COS [19].

## Methods/design

### Registration

This study has been registered on the COMET initiative (http://www.comet-initiative.org/studies/details/941).

### Steering Committee

First, we formed a national Steering Committee to initiate and support the development of the COS. The Steering Committee includes five experts who represent various disciplines, such as a researcher of clinical trials of traditional Chinese medicine in cardiology, a researcher and clinician of traditional Chinese medicine in cardiology, a clinician for the combination of traditional Chinese medicine and western medicine in cardiology, a researcher and clinician of western medicine in cardiology, a methodologist. The five team members make up the Steering Committee that will review and confirm the research protocol, identify the preliminary checklist of the reporting outcome set and outcome instrument set, and attend the consensus meeting to facilitate the development of the COS.

### Design

Eight key stages occur in the development of the COS:

Stage 1: a systematic review will be developed to identify currently reported outcomes and traditional Chinese medicine syndromes in clinical trials of NVAF
Stage 2: patients’ semi-structured interviews will be conducted to fill gaps in potential outcomes
Stage 3: traditional Chinese medicine syndrome names will be identified from medical records
Stage 4: a dataset of traditional Chinese medicine syndrome names will be developed
Stage 5: the investigation of traditional Chinese medicine syndromes will be conducted from a cross-sectional study
Stage 6: two rounds of Delphi surveys will be conducted
Stage 7: a consensus meeting will be held to develop a COS
Stage 8: recommendations of OMIs that should be used in the COS will be developed

**Fig. 1:**
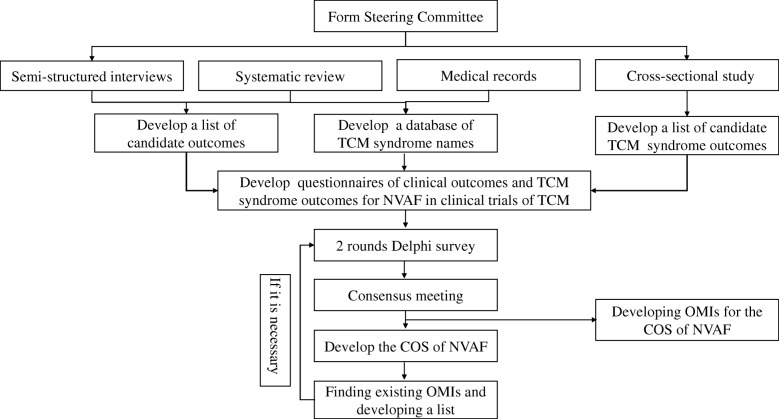
The flowchart of this study. Legend: *TCM* traditional Chinese medicine, *NVAF* non-valvular atrial fibrillation, *OMIs* outcome measurement instruments, *COS* core outcome set

A flowchart of the development of the COS and OMIs is shown in Fig. 1.

### Stage 1: systematic review

A systematic review will be conducted to identify currently reported outcomes and traditional Chinese medicine syndromes in clinical trials of NVAF. In clinical trials and clinical practice, integrative medicine plays an important role in China. Therefore, it is important to include the trials of Western medicine for NVAF to develop a comprehensive outcome list.

#### Search strategy

For English databases, we will search PubMed, the Cochrane Library and the Web of Science. For Chinese databases, we will search the Wanfang database, the China National Knowledge Infrastructure (CNKI), and SinoMed. The trials published from January 2015 to June 2017 will be included. The search strategy of English databases is shown in Additional file 1. The languages are restricted to English and Chinese.

#### Inclusion criteria

The inclusion criteria include the following:

1. Any type of clinical trials such as randomized controlled trials and observational studies
2. For randomized controlled trials, patients with NVAF who accept interventions include any type of Chinese herbal medicine or western medicine, and any type of comparisons are eligible
3. For observational studies, patients with NVAF who accept any type of Chinese herbal medicine or western medicine treatment are eligible
4. Outcomes: all reported outcomes
5. The treatment duration is ≥ 4 weeks
6. The number of participants is ≥ 50 in clinical trials

#### Exclusion criteria

The exclusion criteria include the following:

1. The aim of clinical trials is to investigate the outcome of complications of NVAF (such as disease caused by NVAF)
2. Studies with a primary aim of assessing the mechanism of drug action or pharmacokinetics
3. Full text cannot be acquired

#### Data extraction

Two reviewers will independently assess the titles and abstracts from searches. Then the full texts of the potential articles will be retrieved and assessed for further identification. The data will be extracted independently from included articles by two reviewers. Any disagreement on the eligibility of included articles will be resolved through discussion.

The extracted data include the first author’s name, publication time, number of participants, outcomes (if stated, the primary and secondary outcomes will be identified), the definition of outcomes, outcome measurement instruments, measurement time (intervention duration and follow-up time), and traditional Chinese medicine syndromes (if provided). Any disagreement will be discussed by consulting another investigator.

#### Data analysis and presentation

After the data have been extracted, a list of candidate outcomes will be developed. Two researchers will aggregate the overlapped outcomes together and achieve consensus if necessary. For example, Death, death from any cause, mortality, death within 2 h of the start of exposure to the study drug, overall mortality, total mortality, all causes of death, all causes of mortality will be aggregated as “all-cause mortality.” Then, two authors will group the outcomes together into appropriate outcome domains. The outcome domains and their outcomes will be reviewed by the Steering Committee.

The number of outcomes belonging to an outcome domain will be calculated. The frequency of each individual outcome will be assessed, and the measurement time of each individual outcome will be documented.

For the traditional Chinese medicine syndromes, two researchers will extract every syndrome’s “location of disease” and “feature of disease” independently. Any disagreement will be discussed to achieve consensus.

### Stage 2: patients semi-structured interviews

In previous COS studies, semi-structured interviews have been used to effectively acquire the patients perspective [21, 22]. It is important and necessary to acquire patients opinion in treating NVAF. In China, many patients have not received medical education and there are obstacles for them to understand medical terms; therefore, we prefer inviting the patients to attend semi-structured interviews to obtain their opinions about outcomes of NVAF that should be measured in a clinical trial.

#### Inclusion criteria

Patients aged 50 to 75 years with NVAF and an experience of Chinese herbal medicine treatment will be recruited from Dongzhimen Hospital, Beijing University of Chinese Medicine and Guang’anmen Hospital, China Academy of Chinese Medical Sciences.

#### Exclusion criteria

Patients with a serious medical history who cannot participate in any type of clinical trial and patients with serious mental problems or psychosis will be excluded.

#### Sampling

The sample size of patients recruited for the semi-structured interviews will be 30, which can achieve the objective of saturation from the experience of other similar research projects [23]. However, the final sample size may change; if there are new opinions, the sample size will be increased.

A diversity of age group, gender, AF classification, treatment history, anticoagulant treatment will be included in the purposive sampling. We will recruit patients in accordance with the purposive sampling matrix (Table 1).

**Table 1:** Purposive sampling matrix of patients for semi-structured interviews

Age (years)	Sample size	Gender		AF classification			Treatment history				Anticoagulant treatment	
		Male	Female	Paroxysmal	Persistent	Permanent	Surgery	Pacemaker	Western medicine	Chinese herbal medicine	Anticoagulant	Non-anticoagulant
50–59	10	5	5	4	3	3	2	2	3	3	5	5
60–69	10	5	5	4	3	3	2	2	3	3	5	5
70–79	10	5	5	4	3	3	2	2	3	3	5	5

*AF* atrial fibrillation

#### Recruitment and data collection

Potential participants will be approached at the inpatient ward or outpatient department in the two hospitals at the time of their hospitalization or appointment. An investigator who is trained in qualitative research methods (Qiu RJ) will explain the study to the patients. The patients will be given separate written information sheets to read. Then, the researcher will ask them if they agree to the interview. If so, an informed consent will be signed. Then, a face-to-face conversation will be conducted in the clinical research subject reception room of the two hospitals. It will be made clear to the patients that they can withdraw at any time. Socioeconomic and demographic information as well as disease classification will be collected.

#### Topic guide

All of the semi-structured interviews will be audio-recorded. The interview is based on a topic guide that includes the major aspects of NVAF. The questions in the topic guide are shown in Table 2. The topic guide will be piloted and updated if necessary. The questions will be translated into Chinese. Each participant will have 20 to 30 min to talk with the researcher.

**Table 2:** Questions in the semi-structured interviews for patients

Number	Questions
1	When was the NVAF diagnosed?
2	What type of inconvenience do you have after being diagnosed with NVAF?
3	What type of treatment did you receive after suffering from NVAF?
4	What effect do you want to achieve through treatment?
5	What type of inconvenience does the treatment bring to you?
6	What is the most important outcome for you?

*NVAF* non-valvular atrial fibrillation

#### Data analysis

The analysis will occur concurrently with the data collection. All of the interviews will be transcribed verbatim. We will use qualitative analysis software (NVivo 11, QSR International Pty Ltd., Burlington, MA, USA) to import the recordings. We will use framework methodology to analyze the data, which includes familiarization, developing a thematic framework, indexing, devising thematic charts, mapping, and interpreting [24]. Then, narrative explanations of the effects of NVAF and treatments on the patients’ lives will be interpreted by the process of constant comparison to identify outcome domains that are important to patients [25]. Then, two researchers and the Steering Committee will identify and review, respectively, whether these outcome domains are new, which are different from the systematic review. If they are different, they will be added to the list of candidate outcomes.

### Stage 3: traditional Chinese medicine syndrome names will be identified from medical records

Traditional Chinese medicine syndromes are characteristic of, and the essence of, traditional Chinese medicine. Different patients show diverse syndromes because of different constitutions or symptoms. Traditional Chinese medicine syndromes are difficult to be assessed because the traditional Chinese medicine syndrome names are nonstandard [26, 27]. So it is important to standardize traditional Chinese medicine syndrome names in the development of a COS for clinical trials of traditional Chinese medicine.

In a retrospective study, we will investigate the cases of hospitalized patients aged 50–70 years diagnosed with NVAF from 1 January 2010 to 1 January 2017 in Dongzhimen Hospital, Beijing University of Chinese Medicine. The traditional Chinese medicine syndromes will be extracted from the medical records. Two researchers will extract every syndrome’s “location of disease” and “feature of disease” independently. Any disagreement will be discussed to achieve consensus.

### Stage 4: develop a dataset of traditional Chinese medicine syndrome names

Traditional Chinese medicine syndrome names and their “location of disease” and “feature of disease” from systematic review and medical records will be developed into a dataset.

### Stage 5: the investigation of traditional Chinese medicine syndromes will be conducted from cross-sectional study

To obtain the distribution of traditional Chinese medicine syndromes and obtain traditional Chinese medicine syndrome names which can achieve consensus, the method of epidemiological investigation, such as cross-sectional study, will be conducted.

#### Inclusion criteria

The inclusion criteria of the cross-sectional study are the same as those for stage 2.

#### Exclusion criteria

The exclusion criteria of the cross-sectional study are the same as those for stage 2.

#### Sampling

For the cross-sectional study, we will use convenience sampling to recruit 120 patients with NVAF in Dongzhimen Hospital, Beijing University of Chinese Medicine and Guang’anmen Hospital, China Academy of Chinese Medical Sciences.

#### Recruitment and data collection

Potential participants will be approached at the inpatient ward or outpatient department in the two hospitals at the time of their hospitalization or appointment. Two PhD. students who majored in traditional Chinese medicine will collect the data of symptoms and signs through four methods of diagnosis-inspection, auscultation and olfaction, interrogation, and palpation [4]. They will explain the study to the patients and give separate written information sheets to them. Then, the patients will be asked if they agree to the study. If so, an informed consent will be signed. Then, the researchers will investigate in the clinical research subject reception room of the two hospitals. It will be made clear to the patients that they can withdraw at any time.

For collecting data of symptoms and signs of patients, a sheet of information collection for four methods of diagnosis is developed. Because there is no NVAF diagnosis in traditional Chinese medicine, we refer to different diagnostic criteria of palpitation, such as different versions of *Internal Medicine of Traditional Chinese Medicine*, *Guiding Principles for Clinical Research of Chinese Medicine New Drugs.* Symptoms and signs are extracted from these diagnostic criteria, then the sheet of information collection, which includes socioeconomic demographic information, symptoms, signs, is developed.

#### Data analysis

Symptoms and signs acquired from the cross-sectional study will be used to calculate the frequency. If the frequency of a symptom or sign is < 5%, it will be removed. Then the last data will be conducted clustering analysis with SPSS 20.

From the results of the clustering analysis, two researchers will discuss the classification of traditional Chinese medicine syndromes. Two researchers will extract every syndrome’s “location of disease” and “feature of disease” independently. Any disagreement will be discussed to achieve consensus.

The characteristics of traditional Chinese medicine syndrome classification from the result of clustering analysis will be compared with the dataset of traditional Chinese medicine syndrome names. If the “location of disease” and “feature of disease” are the same or are similar, the traditional Chinese medicine syndrome names will be used as candidate names. Then a questionnaire of traditional Chinese medicine syndromes is developed.

### Stage 6: Delphi survey

#### Stakeholder selection

Three particular stakeholder groups will be invited to participate in a Delphi survey: clinicians (traditional Chinese medicine clinicians, western medicine clinicians, and integrative medicine clinicians), researchers (traditional Chinese medicine clinical researchers, western medicine researchers, and integrative medicine researchers), and nurses.

#### Sampling strategy of the stakeholders

The clinicians and researchers will be obtained from the membership lists of the Clinical Research Method of Cardiovascular Disease of Professional Committee of Chinese Association of Integrative Medicine and the China Research Institute of China Information Association for Traditional Chinese Medicine and Pharmacy. The nurses should work in the department of cardiology in any tertiary hospital in China. There is no standard method for the sample size calculation in Delphi processes at present [28]; therefore, we will use snowball sampling, which involves asking the experts to forward the invitation to colleagues whom they regard as meeting the inclusion criteria of the study.

The Dongzhimen Hospital Ethics Committee has been consulted and has confirmed that the Delphi survey with clinicians and nurses does not require ethical approval.

#### Inclusion criteria of stakeholders:

1. All of the stakeholders should have a bachelor’s degree, at the minimum
2. All of the clinicians should work in tertiary hospitals
3. There is no restriction in locations of the cities for clinicians and nurses
4. The clinicians and nurses should have a junior professional title or above that
5. Researchers as corresponding authors or co-authors should have published at least one article on clinical trials for NVAF in any field (such as internal medicine, surgery, or traditional Chinese medicine)

#### Delphi round 1

The list of candidate outcomes and traditional Chinese medicine syndromes will be separately developed questionnaires. The candidate outcomes questionnaire will be sent to all of the stakeholders by email. The participants will be asked to score all of the items with a 9-point scale in which 1 to 3 means “not important for inclusion in the COS,” 4 to 6 means “important but not critical for inclusion in the COS” and 7 to 9 means “critical for inclusion in the COS” [29]. There are two open questions at the end of the Delphi round 1 questionnaire: (1) What are your suggestions for this questionnaire? and (2) Which outcomes do you think are important, but are not listed in this questionnaire?

The traditional Chinese medicine syndromes questionnaire will be sent by email to stakeholders who have a background in traditional Chinese medicine or integrative medicine education. The participants will be asked to choose a suitable name for every classification of traditional Chinese medicine syndromes from the candidate traditional Chinese medicine syndrome names. Then they will be asked to score all of the symptoms and signs belonging to every classification of traditional Chinese medicine syndrome using a 9-point scale. We will not consult other stakeholders in developing the standard traditional Chinese medicine syndrome names because it is difficult for them to understand traditional Chinese medicine theory and traditional Chinese medicine syndromes.

The participants will be asked to respond with their names and locations. The Steering Committee membership will also be invited to participate in the Delphi survey. We will send a personalized email outlining the project to stakeholder groups and invite them to complete Delphi round 1 within 2–3 weeks.

#### Data analysis of Delphi round 1

If there are additional outcomes recommended by stakeholders, they will be submitted and reviewed by the Steering Committee to determine if they are new. We will analyze the responses of round 1 and calculate the frequencies for the response options of each item as a whole. Each stakeholder group’s score distribution will be analyzed. All of the outcomes will be carried forward to round 2.

#### Delphi round 2

The individuals who participated in Delphi round 1 and finished the survey will be invited to participate in round 2. The number of participants and the distribution of scores for each outcome will be shown to every participant in round 2. They will be shown the scores of each item and the distributions of scores from their own stakeholder group. The participants will be asked to consider the other stakeholders’ responses and to re-score the items. If any of the participants change their scores, they will be asked to provide a reason. They will have 2–3 weeks to complete the survey. We will send a personalized email to remind the participants completing the survey.

#### Data analysis of Delphi round 2

After completion of Delphi round 2, we will analyze the responses of round 2 and calculate the frequencies for the response options of each outcome as a whole. Each stakeholder group’s score distribution will be analyzed. We will examine the number of participants completing rounds 1 and 2 for assessing a potential attrition bias. The changes of scores between the two rounds and the reasons for changes will be summarized.

### Stage 7: consensus meeting

The final phase of developing a COS is a face-to-face consensus meeting. All of the members of the Steering Committee will attend the meeting. They should invite an additional 20 participants who have a master’s degree and deputy chief physician professional title or higher to attend the consensus meeting. The participants in the consensus meeting will include clinicians, researchers, nurses, and methodological experts, regardless of whether or not they participated in the Delphi survey. A total of 25 participants should attend the consensus meeting.

At the consensus meeting, the results of the scores of each outcome from round 2 of the Delphi survey will be presented. The number of outcomes that have achieved consensus by all of the stakeholder groups or by any of the stakeholder groups will be reported. The outcomes that have achieved “consensus in” by all of the stakeholder groups will be included in the COS. The outcomes that have achieved “consensus out” by all of the stakeholder groups will be excluded. The remaining outcomes will be discussed by any of the participants of the consensus meeting. Then, the participants will be asked to score all of the outcomes that have not reached a consensus by an anonymous method at the meeting. Then, the outcomes that have achieved consensus will be included in the COS of NVAF. The definition of the consensus are shown in Table 3 [24].

**Table 3:** Definitions of consensus

Consensus classification	Description	Definition
Consensus in	The outcome should be included in the core outcome set	70% or more of the participants scored outcome as 7 to 9, and < 15% of the participants scored the outcomes as 1 to 3
Consensus out	The outcome should not be included in the core outcomes set	70% or more of the participants scored the outcome as 1 to 3, and < 15% of the participants scored the outcome as 7 to 9
No consensus	Uncertainty of the importance of outcome	Anything else

After developing the final COS, the time point of measurement for every included outcome will be discussed and recommended at the consensus meeting.

### Stage 8: developing OMIs for the COS

After completing the COS of NVAF, the study will be continued to select the OMIs for the included outcomes.

The objective outcomes will be recommended at the consensus meeting based on the results of a systematic review from stage 1.

The subjective outcomes will be recommended on the basis of the guidelines that are included in the paper of “How to select outcome measurement instruments for outcomes included in a ‘core outcome set’ – a practical guideline” [20], which includes four steps:

Step 1: conducting systematic reviews to find existing OMIs for measuring the outcomes in the COS
Step 2: two researchers will evaluate the quality of the measurement properties and the feasibility of the identified OMIs with the COSMIN (COnsensus-based Standards for the selection of health Measurement INstruments) Checklist independently, and any inconformity will be discussed in consultation with a third investigator [30]
Step 3: two rounds of Delphi surveys will be conducted by different stakeholder groups. The methods of selecting the stakeholder groups and the Delphi survey are the same as for stage 6. There will be a semi-structured interview with patients to obtain their perspectives on selecting the OMIs. The method of the semi-structured interview is the same as for stage 2
Step 4: if any of the OMIs do not achieve consensus, different stakeholder group representatives will be invited to attend another consensus meeting to develop consensus in selecting the OMIs for each outcome. The method of the consensus meeting is the same as for stage 7

## Discussion

There are many problems in outcomes’ reporting of clinical trials of traditional Chinese medicine. It is very important to develop a standardized and minimum outcome set, which can achieve consensus by different stakeholders so that it can be applied in clinical trials and systematic reviews to help translate the results into high-quality evidence. As described in the COMET initiative, the COS does not mean that researchers should only report the outcomes that are described in the COS. Rather, there is an expectation that the core outcomes will be collected and reported, making it easier for the results of trials to be compared, contrasted and combined as appropriate while researchers continue to explore other outcomes (http://www.comet-initiative.org.).

**Fig. 2:**
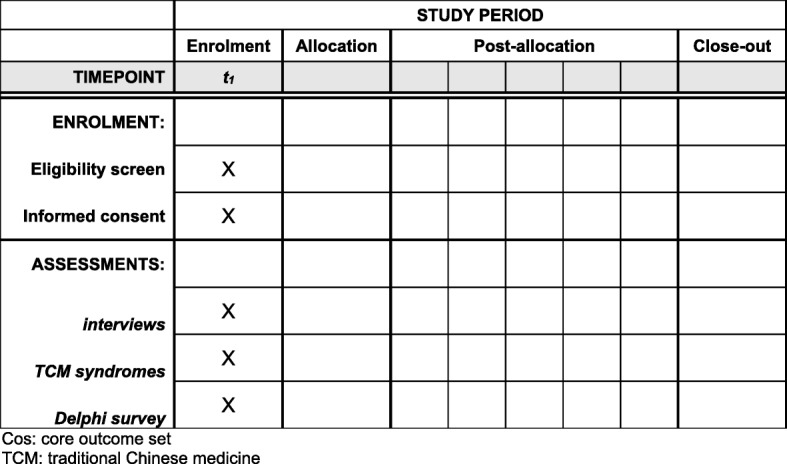
Standard Protocol Items: Recommendations for Interventional Trials (SPIRIT) Figure

NVAF is common in aging patients in China. Many patients prefer to undergo traditional Chinese medicine treatment or integrative medicine treatment, and a large number of clinical trials are currently being conducted for the treatment of NVAF. However, the effectiveness and safety of Chinese herbal medicine remains unclear, which is related to nonstandardized outcome reporting. We hope that our work will improve the quality of traditional Chinese medicine clinical trials in NVAF via limiting the heterogeneity of different outcome reporting to ensure the comparability of the effects and the synthesis of the results in meta-analyses in the future. Meanwhile, if researchers select a COS as the standard of outcome reporting in all types of clinical trials, it can decrease the risk of reporting bias to some extent. The Standard Protocol Items: Recommendations for Interventional Trials (SPIRIT) Checklist shows the detailed information of the protocol items (Additional file 2). The SPIRIT Figure shows in detail the schedule of enrollment and assessments (Fig. 2).

### Study status

At the time of the revised manuscript submission, this study is inviting experts to attend consensus meeting for the COS.

## Additional files

Additional file 1: The search strategy of the systematic review. (DOCX 13 kb)

Additional file 2: Standard Protocol Items: Recommendations for Interventional Trials (SPIRIT) 2013 Checklist: recommended items to address in a clinical trial protocol and related documents. (DOCX 49 kb)

## Abbreviations

COMET: Core Outcome Measures in Effectiveness Trials
COS: Core outcome set
COSMIN: COnsensus-based Standards for the selection of health Measurement INstruments
NVAF: Non-valvular atrial fibrillation
OMIs: Outcome measurement instruments
