# Supplementary material for: Development of a core outcome set (COS) and selecting outcome measurement instruments (OMIs) for non-valvular atrial fibrillation in traditional Chinese medicine clinical trials: study protocol
Source: Trials. 2018 Oct 5;19:541. doi: 10.1186/s13063-018-2904-0 (PMC6173842; doi:10.1186/s13063-018-2904-0)
Supplement: Supplementary file 1 — The search strategy of the systematic review. (DOCX 13 kb) [file 13063_2018_2904_MOESM1_ESM.docx]

**Search strategy for Chinese herbal medicine**

Pubmed:

#1 "Atrial Fibrillation"[Mesh]

#2(((((auricular fibrillation[Title/Abstract]) OR atrial fibrillation[Title/Abstract]) OR AF[Title/Abstract]) OR palpitation[Title/Abstract]) OR cardiopalmus[Title/Abstract]) OR palpitate[Title/Abstract]

#3 (((((random*[Title/Abstract]) OR blind*[Title/Abstract]) OR control*[Title/Abstract]) OR observational study[Title/Abstract]) OR clinical trial*[Title/Abstract]) OR observational studies[Title/Abstract]

#4 #1 or #2

#5 #4 and #3

#6 (((surgery[Title/Abstract]) OR operation[Title/Abstract]) OR ablation[Title/Abstract]) OR RFCA[Title/Abstract]

#7 #5 not #6

Date – Publication:2015/1/1-2017/6/1

Cochrane Library:

#1 auricular fibrillation:ti,ab,kw or atrial fibrillation:ti,ab,kw or AF:ti,ab,kw or palpitation:ti,ab,kw or cardiopalmus:ti,ab,kw Publication Year from 2015 to 2017 (Word variations have been searched)

#2 surgery:ti,ab,kw or operation:ti,ab,kw or ablation:ti,ab,kw or RFCA:ti,ab,kw Publication Year from 2015 to 2017 (Word variations have been searched)

#3 #1 not #2

Web of science：

TS=((auricular fibrillation OR atrial fibrillation OR AF OR palpitation OR cardiopalmus OR palpitate) AND (random* OR blind* OR control* OR observational study OR clinical trial* OR observational studies)) NOT TS=(surgery OR operation OR ablation OR RFCA)

**Search strategy for non-herbal medicine**

Pubmed: (n=3)

#1 "Atrial Fibrillation"[Mesh]

#2 (((((auricular fibrillation[Title/Abstract]) OR atrial fibrillation[Title/Abstract]) OR AF[Title/Abstract]) OR palpitation[Title/Abstract]) OR cardiopalmus[Title/Abstract]) OR palpitate[Title/Abstract]

#3 (((((((dietary supplement*[Title/Abstract]) OR acupuncture[Title/Abstract]) OR massage[Title/Abstract]) OR moxibustion[Title/Abstract]) OR cupping therapy[Title/Abstract]) OR Tai Chi[Title/Abstract]) OR qigong[Title/Abstract]) OR qi gong[Title/Abstract]

#4 (((((random*[Title/Abstract]) OR blind*[Title/Abstract]) OR control*[Title/Abstract]) OR observational study[Title/Abstract]) OR clinical trial*[Title/Abstract]) OR observational studies[Title/Abstract]

#5 #1 or #2

#6 #5 and #4 and #3

Cochrane Library: (n=85)

#1 auricular fibrillation:ti,ab,kw or atrial fibrillation:ti,ab,kw or AF:ti,ab,kw or palpitation:ti,ab,kw or cardiopalmus:ti,ab,kw Publication Year from 2015 to 2017 (Word variations have been searched)

#2 dietary supplement*:ti,ab,kw or acupuncture:ti,ab,kw or massage:ti,ab,kw or moxibustion:ti,ab,kw or cupping therapy:ti,ab,kw or Tai Chi:ti,ab,kw or qigong:ti,ab,kw or qi gong:ti,ab,kw Publication Year from 2015 to 2017 (Word variations have been searched)

Web of science: (n=30)

#1 TS= auricular fibrillation OR atrial fibrillation OR AF OR palpitation OR cardiopalmus OR palpitate

#2 dietary supplement* OR acupuncture OR massage OR moxibustion OR cupping therapy OR Tai Chi OR qigong OR qi gong

#3 TS= random* OR blind* OR control* OR observational study OR clinical trial* OR observational studies

#4 #1 AND #2 AND #3

2015-2017
